# Is physician location sensitive to changes in patients’ financial responsibility?

**DOI:** 10.1080/15140326.2022.2041158

**Published:** 2022-03-09

**Authors:** Marion Aouad

**Affiliations:** Department of Economics, University of California, Irvine, California, US

**Keywords:** Physician agency, health insurance, insurance design, insurance reform

## Abstract

This study examines how changes to patients’ financial responsibility affect physicians’ behavior. This is achieved by examining a health insurance reform that changes patients’ relative financial responsibilities for a medical service that can be received at one of two locations. In particular, this study examines how physicians’ treatment location decisions change after the reform. This study finds that physicians who previously work across the two locations are increasingly observed working at the location that becomes cheaper for patients. Thus, physicians’ responsiveness to new policies may be an important lever by which certain demand-side health insurance reforms successfully operate.

## 1. Introduction

Health insurance reforms often take the form of demand-side focused policies. For example, over the last ten years there has been a growth in the availability of high-deductible health plans (Brot-Goldberg, Chandra, Handel, and Kolstad (2017) and Cohen and Zammitti (2018)) and other policies, such as narrowed health insurance networks or reduced insurer contributions to cost-sharing (e.g., Gruber and McKnight (2016) and Robinson and Brown (2013)). Common to these reforms is that the financial burden is placed on patients, who increasingly bear greater financial responsibility for the medical care received. However, every treatment decision involves a physician. Yet, there is limited empirical evidence on how physicians behaviors are affected by demand-side health insurance reforms. Existing evidence suggests that physicians are either generally unaware of the prices faced by patients (Alexander, Casalino, and Meltzer (2005)) or that they only respond to large, widely publicized price changes (Carrera, Goldman, Joyce, and Sood (2018)). Thus, it remains unclear how physicians’ behaviors are affected by demand-side health insurance reforms, particularly those that are less salient and only affect a subset of patients.

This study examines how physicians’ respond to changes in patients’ financial incentives by examining how physicians location decisions change in response to a health insurance reform that only directly affects patients’ financial responsibility. This is achieved by exploiting the introduction of a *reference pricing* health insurance reform introduced by the California Public Employees’ Retirement System (CalPERS). Reference pricing changes patients’ financial responsibility (cost-sharing) across higher-priced and lower-priced medical locations: if patients visit a hospital (i.e., a higher-price setting), they now face a set maximum reimbursable amount; if they visit an Ambulatory Surgery Center/ASC (i.e., a lower-price setting), their cost-sharing is unchanged and no maximum reimbursable amount is set. Additionally, physicians’ immediate financial reimbursement is *not* affected by the reform and remains similar across ASCs and hospitals. Thus, by leveraging the changes to patients’ financial responsibility stemming from the introduction of this health insurance reform, this study is able to examine how physicians respond to demand-side health insurance reforms.

To examine this question, I utilize patient-physician linked medical claims data. From this a balanced physician panel is formed. Also observed is a comparable control group of patients who do not experience any changes to their insurance reimbursement and for whom the physician is compensated the same fee. Using Difference-in-Difference models, I exploit the as-good-as-random timing in the introduction of the reform to estimate the impact on physicians’ likelihood of treating a CalPERS patient at an ASC relative to that of a control group patient.

Since it may take time to negotiate contracts that allow a physician to work in a new location, physicians’ pre-existing location affiliations likely affect their responsiveness to the reform. Therefore, I focus on physicians who previously spent time at both ASCs and hospitals, referring to these physicians as “Splitters” since they split their time across both settings (David and Neuman (2011)). This approach is taken because these physicians likely have the most capacity to easily switch locations due to pre-existing networks and institutional arrangements (Rizzo and Goddeeris (1998)). To further understand the behavioral effects of the reform, I also estimate changes in the observed number of days spent at an ASC to understand if the reform simply causes physicians to work more hours, or instead reallocate more hours to the setting that is now cheaper for patients (ASCs).

There exist several studies that evaluate the CalPERS-specific reference program. Many of these studies examine where patients receive their medical treatment after the reform is passed as well as the associated cost-savings from patient movement (e.g., Aouad, Brown, and Whaley (2019), Whaley, Guo, and Brown (2017), Robinson, Brown, Whaley, and Finlayson (2015)). These studies find that after the reform is passed, patients are more likely to visit the ASCs. However, these studies, do not examine the response of physicians to the reform.^1^ Yet, this is a non-trivial question since demand-side reforms are only effective if there exist responsive physicians who are able to facilitate patients’ desire for lower-priced medical care. Furthermore, if there are binding constraints that make it difficult for physicians to alter the location in which they provide treatment, demand-side reforms may be less effective than anticipated.

Thus, the innovation of this paper is that it: i) determines whether demand-side health insurance reforms affect physician behaviors, particularly focusing on physicians observed before the reform is enacted and ii) examines if physician institutions are important considerations when implementing health insurance reforms. For example, in this setting, knowing the share of physicians who can practice at both ASCs and hospitals is important if it takes time for physicians to establish access to ASCs.

The results indicate that physicians are responsive to group-specific prices and respond to the changes in financial incentives faced by patients by moving to the setting that is cheaper for patients. In particular, physicians who work at both ASCs and hospitals are approximately 6.4 percentage points (9 percent) more likely to treat a CalPERS patient at an ASC after the reform is introduced. This could be due either to altruistic (Carrera et al. (2018)) or profit motives (e.g., McGuire (2000)). This study is not able to conclusively differentiate between these two motivations. However, suggestive evidence points to physicians’ personal financial interests as a potential motivating factor. The findings of this study remain important because a physician, in principle, could choose not to move to the location that is cheapest for a patient. As such, this is one of a few studies to show that physicians are mobile across locations, for a given medical procedure, and that a health insurance reform that only affects patients’ cost-sharing can induce treatment location mobility among physicians. In turn, this implies that physicians’ location mobility can serve as a mechanism to reduce health care spending.

The introduction of the reform also coincides with physicians’ increasingly supplying labor at ASCs. In particular, physicians who previously spent time at both ASCs and hospitals prior to the introduction of the policy, are not observed working more days. Rather, they are observed spending a *greater share* of work days at ASCs in the year after the policy is introduced.

This paper makes two contributions to the broader literature. First, it allows for an improved understanding of the factors motivating physicians’ decisions. In particular, I provide strong evidence that changes to patient cost-sharing are relevant. Furthermore, this study shows that price changes that may be less salient to the physician and only impact one group of patients can still affect the actions of physicians. This is consistent with Carrera et al. (2018) who show that physicians change their prescription drug prescribing patterns for widely publicized price decreases, particularly for poorer patients. However, this study differs in that physicians’ “salience” of the cost-sharing change experienced by CalPERS patients is arguably much smaller; the share of patients, observed in the data, who belong to a CalPERS health plan is approximately 11 percent in the pre-reference pricing period.^2^ Instead, the physician agency literature has typically focused on the response of physicians to *immediate* or *direct* financial incentives (e.g., reimbursement differences) and has shown that physicians are responsive (e.g., Ho and Pakes (2014), Clemens and Gottlieb (2014), and David and Neuman (2011)). Yet, non-immediate financial incentives such as own performance information (Kolstad (2013)) and altruism toward lower-socioeconomic status patients (Chen and Lakdawalla (2019)) have also been shown to matter for physicians’ behaviors.

Second, this work adds to the literature on understanding the mechanisms by which demand-side health insurance reforms operate by tracing out physicians responses to the referencing pricing reform. In particular, this analysis shows that changes in the behavior of *existing* physicians are important to the success of this reform.^3^ This is in contrast to the alternative of patients finding completely “new” physicians who were not treating CalPERS patients prior to the reform. The literature has, instead, typically focused on alternative mechanisms including reductions in the quantity of medical care demanded by patients (Manning et al. (1987)).

Lastly, this paper has important policy implications. Given that health care spending makes up nearly 20 percent of GDP in the US (CMS (2020)), a number of new policies have been enacted in order to reign in the growth of health care spending. Such programs include the Hospital Readmissions Reduction Program, which aims to affect the behavior of health care providers by affecting their reimbursement (Gupta (2021)). Yet, this work shows that another way to affect health care supplier’s behaviors, which affects aggregate medical spending, is to directly affect how much patients pay; this stands in contrast to only adjusting health care providers’ reimbursements. This insight stems from this paper’s findings, which show that physicians are willing to relocate their treatment location, from traditionally higher-price settings (hospitals) to lower-price settings (ASCs), after a change to patients’ cost-sharing. This is the first study, known to the author, to demonstrate such a finding, which has very important policy implications as it relates to mitigating the growth in health care spending.

The rest of the paper will proceed as follows: Section 2 discusses the reference pricing policy and Section 3 discusses the conceptual framework underlying physicians’ location decisions. Section 4 discusses data. Section 5 discusses the methodology and empirical specifications while Section 6 presents the results. The paper is concluded in Section 7.

## 2. Policy and institutions

### 2.1. Policy

Reference pricing was first introduced in 2011 for the California Public Employees’ Retirement System (CalPERS), the second largest public purchaser of health benefits in the United States (CalPERS (2019)).^4^ The program was introduced in 2011, among PPO health insurance beneficiaries receiving hip and knee replacement surgeries. In 2012, the program was expanded to colonoscopy, arthroscopy, and cataracts medical procedures. This analysis focuses solely on colonoscopies because it is the most common procedure for which patients receive treatment among the procedures subjected to reference pricing.

The program incentivizes CalPERS patients receiving colonoscopies to choose ASCs over hospitals by changing the relative prices patients face across these two, common medical settings.^5^ ASCs are smaller out-patient health care facilities, that mainly specialize in a select number of procedures. This is in contrast to hospitals, who provide a variety of medical services and have inpatient and emergency room facilities. Most ASCs are owned by physicians/physician-groups or with other investors (Bian and Morrisey (2007)).

Prior to the reform, CalPERS patients who visited either an ASC or hospital were subject to the standard cost-sharing of their health insurance. After the reform, a maximum reimbursable amount of $1500 is set for CalPERS patients who continue to visit hospitals after the policy is introduced.^6,7^ At the hospital, every dollar of the total price above this reference price is 100 percent the responsibility of the patient and does not go toward their annual out-of-pocket maximum nor their annual deductible. However, if a patient visits an ASC for their medical care, after reference pricing is introduced, patients do not face a reference price and incur the standard cost-sharing they faced in the pre-reference pricing period.^8^ Note that the cost-sharing amount is based on the date of the medical appointment and not the date the appointment is made.

Anthem BlueCross (Anthem) of California administers the health benefits for the CalPERS PPO members and for other Californians. These other California PPO members for whom Anthem also administers health insurance benefits form the control group for this study. Control group members face the same price as CalPERS members at any given health care facility/physician but are not subject to the reference pricing policy.^9^ Additionally, reference pricing does not affect physicians’ reimbursement. Physicians’ reimbursement remains similar at both the ASC and hospital and is the same when treating either a CalPERS or control patient.

## 3. Conceptual framework

To motivate the physician’s location decision process, I model physicians’ utility from treating patients at either an ASC or hospital as a discrete choice, employing the common physician utility framework (e.g., Ellis and McGuire (1986), McGuire (2000)). I further assume that medical location decisions are ultimately decided upon by the physician (e.g., Chen and Lakdawalla (2019)).^10^

Let $U_{j}^{i}=\alpha B^{i}+(1-\alpha )\pi^{i}+\varepsilon^{i}wherej\in \{ASC,\;Hospital\}\;and\;\alpha \in [0,1]$

$U_{j}^{i}$ is the utility for the physician from performing a procedure in medical setting j for patient i. $B_{j}^{i}$ is the benefit or utility to patient *i* from having a procedure done in facility *j*. $\pi_{j}^{i}$ is the profit received by the physician from performing a medical procedure for patient i at facility j. Note that physicians’ immediate profit from either facility is nearly identical and has not been affected by the reform. *α* represents the weight that the physician puts on their own profit versus the weight that they put on the patient’s benefit. It is assumed to be greater than zero based on prior studies (Godager and Wiesen (2013), Carrera et al. (2018)). $\varepsilon_{j}^{i}$ captures the effect on utility not captured in $B_{j}^{i}$ or $\pi_{j}^{i}$. For example, one can assume that it reflects unobserved tastes of the physician.

Let *p^i^*(*j*∣*k*) represent the probability that a physician treats patient *i* at facility *j*, at time *t*, given that the patient’s desired treatment location is *k*. This probability arises from the joint interaction between physicians and patients and may not necessarily equal one (De Jaegher and Jegers (2001)). As an example, *p^i^*(*j*∣*k*) could reflect the probability that physicians treat a patient at an ASC conditional on the patient desiring treatment at an ASC, at time *t*. Thus, the physician’s expected utility from working with patients at facility *j* at time *t* is:^11^ $E{\left(U_{j}\right)}_{t}=\frac{1}{R}\sum_{i=1}^{R}p_{t}^{i}(j|k\in \{ASC,Hosp\})\left\{\alpha B_{j,t}^{i}+(1-\alpha )\pi_{\;j,t}^{i}\right\}$

As shown, the physician’s expected utility from working at facility *j*, at time *t* is both a function of the expected benefit to the patient, as perceived by the physician, and the physician’s expected profit. Thus, if the share of CalPERS patients treated at an ASC increases after the policy is introduced (i.e., *ΔE*(*U_ASC_*) > 0), this could be due to either changes to patient benefit, *B*, (owing to changes in patient cost-sharing) or to changes in physicians’ expected profit. Unfortunately, this study is not able to conclusively distinguish between these two motives due to data limitations. However, the latter case is particularly likely if there may be additional returns to physicians beyond the direct reimbursement from working at an ASC (e.g., additional equity returns from ownership stakes in ASCs). This is investigated in a further section.

## 4. Data

Data consists of Anthem PPO medical claims for CalPERs and control group members (i.e., Anthem PPO members who do not belong to CalPERS) who have a colonoscopy between 2009 and 2013. The dataset is ideal for this analysis given its size. By focusing on colonoscopies, a relatively common procedure, the results may also be more likely to generalize to a wider population since colonoscopies are recommended for adults over the age of 50 years for preventative/screening purposes (US Preventative Task Force (2016)). Also, approximately 15 million colonoscopies were performed in 2012 (Joseph et al. (2016)), which is more than four times the estimated number of arthroscopy and cataracts procedures.

Observed in this dataset are the physicians performing the colonoscopies and the locations in which the procedures are performed: ASC or hospital. From this, a balanced panel of physicians who are observed performing a colonoscopy, with either a control or CalPERS patient, in all years of 2011, 2012 and 2013, is formed. This structure is chosen due to the nature of claims data, which, by design, capture the health care utilization of patients. Thus, in order to capture the location decisions of physicians and patients over time, the sample is restricted to physicians who can be observed in the years of interest.

The years 2011–2013 are selected to ensure that the largest number of physicians are included in the sample and to capture patient-physician observations before and after the reform, which occurs in 2012. Additionally, if a physician appears in all years of 2011, 2012 and 2013, observations for that physician from 2009 and 2010 are also used, if available. Data are also limited to solo-practicing physicians. (i.e., physician groups are excluded from the analysis).^12^ This is done in order to accurately categorize physicians’ into the appropriate work categories/types. This removes approximately 42 percent of total observations over the 2009–2013 period. However, physician groups are later included as part of robustness checks.

The analysis then focuses on physicians who split their time at both ASCs and hospitals prior to the reform (i.e., “Splitters”), borrowing from the work of David and Neuman (2011).^13^ These physicians are observed with patients at both ASCs and hospitals prior the reference pricing reform. Specifically, the physicians who form the basis for thisanalysis are observed with a patient between 5 percent and 95 percent of the time at an ASC in 2011.^14^ Approximately 42 percent of physicians observed in 2011 fall under this definition.

Linked to each physician observation are the characteristics of each patient treated. Patient-linked data includes information on the patient’s age (between 18 and 64 years), sex, an indicator for whether or not the patient has a medical intervention during the colonoscopy (e.g., a polyp removed) as well as the Charlson Comorbidity score, which is used as a proxy for the patient’s health status (Charlson, Pompei, Ales, and MacKenzie (1987)).^15^ Also included is data on the physician’s reimbursement (physician fee) and the total price (facility fee) paid by the patient for the medical treatment.

Of note, the resulting sample consists of physicians selected by patients. However, it is still informative for the analysis. In particular, approximately 85 percent of observations come from physician who are observed before and after the reform (i.e., 2011–2013). This implies that observed results will be driven by the majority of the entire physician sample.^16^ Additionally, the sample of physicians includes those who are visited by either CalPERS patients or a control group patient, implying that physicians’ inclusion in the sample is not exclusively dependent on their responsiveness to the reference pricing policy.

Table 1 and Table 2 provide summary statistics in the three years prior to the reform and the two years after the reform. The data show that the characteristics of the patients treated by physicians remain relatively steady over time. For example, the share of patients who are male is relatively steady around 46 percent while the average age of patients is approximately 53 years across all physician types. However, the share of time that physicians spent at the ASC in a given year is increasing over time. This change is greatest when treating CalPERS patients; the share of CalPERS patients seen at ASCs is nearly 20 percent higher between 2013 and 2011, versus an increase of approximately 8 percent when treating a control patient.

Additionally, average physician reimbursement is similar at both ASCs and hospitals in the years leading up to the reform. This is consistent with the fact that physician reimbursements are the same for physicians who see patients either at an ASC or a hospital among Medicare beneficiaries (Munnich and Parente (2018)). This is also in line with the reference pricing policy, which did not affect physician reimbursements.

## 5. Methodology and empirical specification

To examine physicians’ location response to the changes in patient cost-sharing, this analysis exploits the *as-good-as-random* introduction of the insurance reform and the accompanying changes to patient cost-sharing in order to determine how physicians’ location decisions are affected. In particular, reference pricing is introduced with limited notice to beneficiaries^17^ and does not directly affect physician reimbursement. This program design lends itself to the use of a Difference-in-Difference analysis, which relies on the validity of a parallel trends assumption. The parallel trends assumption is that in the absence of the reform, physicians’ rate of treating CalPERS patients at ASCs would have trended similarly to their rate of treating control group patients at ASCs. This assumption is likely to hold given that physician reimbursement has not changed and does not vary across the two patient groups. Further, both CalPERS and control patients are demographically similar.

### 5.1. Main empirical specification

The main empirical specification used is a Difference-in-Difference strategy and is presented in Equation 1:

(1) $$1(ASC{)}_{ki}=\gamma_{{}_{0}}+\gamma_{{}_{1}}\times Post_{{}_{i}}+\gamma_{{}_{2}}\times Treat_{{}_{i}}+\gamma_{{}_{3}}\times Post_{{}_{i}}\times Treat_{{}_{i}}+X_{i}^{\prime }\gamma +\alpha_{k}+u_{ki}$$

*γ*_3_ is the primary coefficient of interest and indicates the increased probability of a physician treating a CalPERS patient at an ASC, relative to a control patient, after the introduction of reference pricing. 1(*ASC_ki_*) is a binary variable that takes the value one if physician *k* is observed treating patient *i* at an ASC and is zero if the physician is observed with the patient at the hospital. *Post_i_* takes the value of one if observation *i* is from the post-reference pricing period and is zero if it is from the pre-period. *Treat_i_* is also binary and is equal to one if the physician treats a CalPERS patient and is zero if the physician treats a control group patient. *X*_*i*_ represent patient covariates that could affect the probability of observing a physician performing a colonoscopy at an ASC, as discussed above.^18^ Also, to account for unobserved time-invariant heterogeneity across physicians, a physician fixed effect, *α_k_*, is included.

Equation 1 is estimated using a linear probability model. Implicit in this analysis is the assumption that any unobserved characteristics of the physician which could influence their response to changes in patient demand are time-invariant and are captured by the physician fixed effect, *α*_*k*_. If this assumption holds, *γ*_3_ can be interpreted as the effect of reference pricing on the location decisions among physicians for whom pre- and post-period claims are observed.

### 5.2. Parallel trends

To test whether physicians similarly treat CalPERS patients and control group patients at ASCs prior to the policy, I estimate the adjusted share of patients seen at an ASC versus at a hospital. I do this by estimating the probability of a physician being observed with a patient at an ASC, controlling for patient characteristics, using Equation 2:

(2) $$1(ASC{)}_{{}_{i}}=\alpha_{{}_{0}}+\sum_{r=-12,r\neq -1}\beta_{{}_{r}}1(T_{{}_{i}}=r)+\sum_{r=-12}^{7}\rho_{{}_{r}}1(T_{{}_{i}}=r)\times 1(CalPERS{)}_{{}_{i}}+X_{i}^{\prime }☒+\varepsilon_{i}$$

The main parameters of interest are *ρ*_*r*_ for the values of *r* ranging between −12 and −1, where *r* represents the number of quarters since reference pricing is implemented. Estimates of *ρ*_*r*_ give the added probability of a CalPERS patient being seen at an ASC by a physician, relative to a control patient *r* quarters since reference pricing is introduced.

While *ρ* does not have to be equal to zero over time, it should be stable in the quarters leading up to reference pricing for the parallel trends assumption to hold. The variable 1(*T*_*i*_ = *r*) takes the value one if the time period observed is *r* quarters away from when reference pricing was introduced and is zero otherwise. 1(*ASC*_*i*_) is equal to one if the *i*^*th*^ patient is treated at an ASC and is zero if it is observed at the hospital. 1(*CalPERS*_*i*_) is equal to one if patient is part of CalPERS and is zero if they belong to the control group. Also included are the vector of patient demographic covariates, *X*_*i*_.

### 5.3. Study limitations

There are two limitations of this analysis. First, I do not observe the choices for all physicians involved in the medical decisions. Instead, I am only able to observe a portion of the physician response to changes in patients’ financial responsibility. In particular, primary care physicians, who are typically the first physicians seen by patients, may change their referral patterns in response to the reform. Unfortunately, primary care physician referrals are not observed. However, any change in the practice patterns of physicians who are observed (e.g., downstream effects) is still an interesting result. Second, I only observe the physician response for those physicians who have “matched” or come to a location agreement with patients over the sample period of consideration. This may lead to observations for only those physicians most amenable to location movement. This may overstate (i.e., upward bias) estimates of physicians’ location mobility if the observed physicians are the most amenable or responsive to changes in patient cost-sharing. This could provide a challenge to external validity if the broader physician population differs. However, the results are still insightful for understanding how a relatively large share of physicians, who are observed prior to the implementation of the policy, respond to the insurance reform.^19^

## 6. Results

In this section I present the results of the parallel trends analysis as well as the main results. I also discuss the motives for physicians’ location movement and the mechanism by which this may occur.

### 6.1. Parallel trends

**Figure 1.**
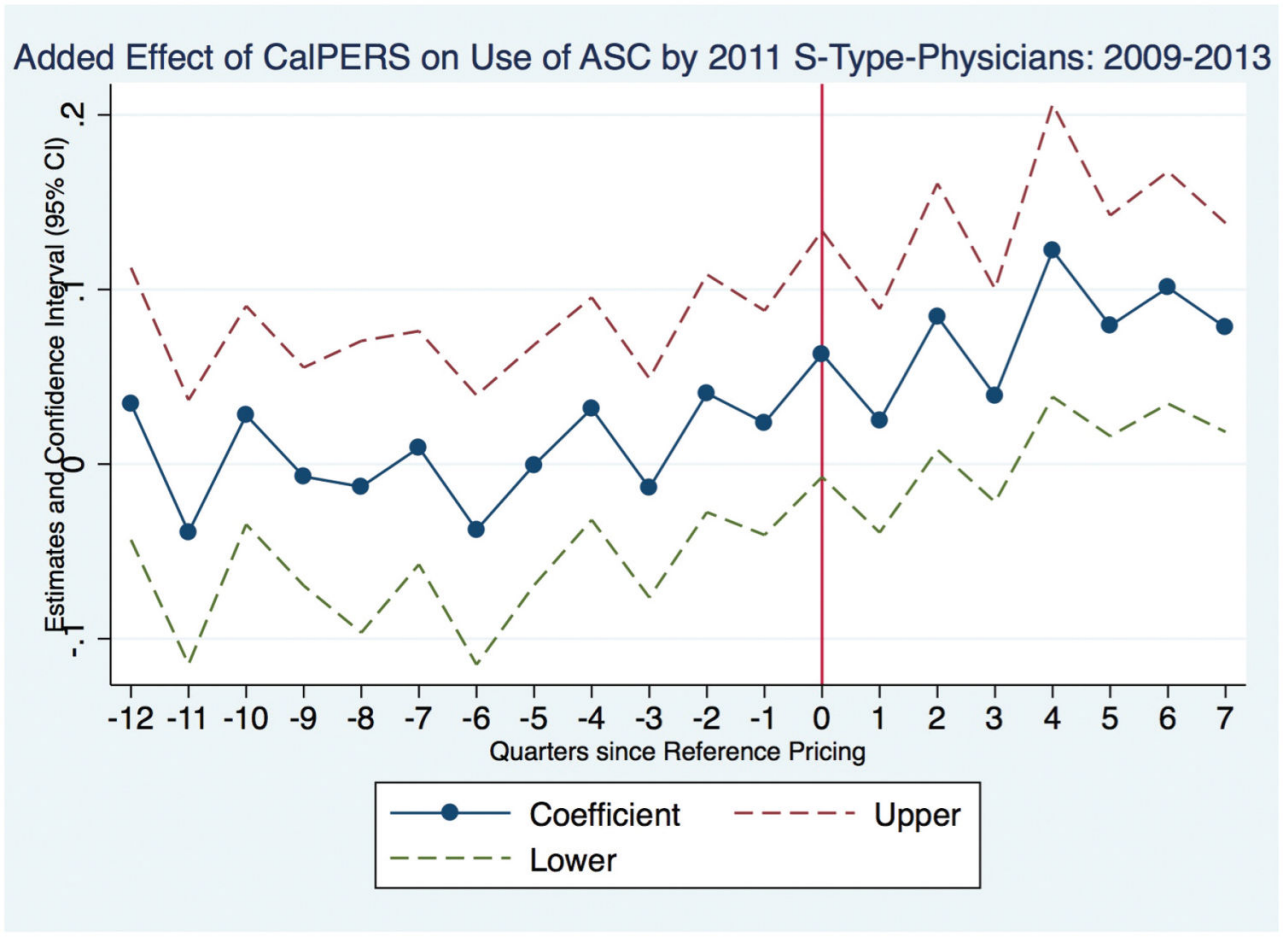
Share of patients treated at an ambulatory surgery center (ASC). This figure presents coefficients estimates of *ρ* from the event study in Equation 2. Each estimate represents the added effect of CalPERS membership on the probability of a patient being treated at an ASC, conditional on receiving a colonoscopy from a Splitter physician (i.e., physicians who work at both hospitals and Ambulatory Surgery Centers in 2011).

Figure 1 presents estimates of *ρ*_*r*_ from estimation of Equation 2 and the standard error of the estimate in each quarter (*r*) since reference pricing. This figure shows the trends in the share of patients being treated at ASCs.

This figure shows that leading up to the introduction of the policy, CalPERS patients are just as likely to be treated at an ASC as a control patient. While Figure 1 exhibits some variability in the share of CalPERS patients seen at an ASC (relative to control patients) in the quarters leading up to reference pricing, this variability is likely driven by the relatively smaller sample size of the CalPERS patient group. Furthermore, the estimates of *ρ*_*r*_ are not statistically different from zero in the quarters preceding reference pricing. Additionally, the figure shows that the probability of a physician treating a CalPERS patient at an ASC increases after the policy is introduced. In particular, one year after the reform, the point estimates for *ρ*_*r*_ are positive and statistically different from zero.

### 6.2. Main results

The main results from Equation 1 are presented in Table 3. The results indicate that the introduction of reference pricing leads to an increase of approximately 6.4 percentage points (9 percent) in the probability of a physician treating a CalPERS patients at an ASC, relative to a control group patient. This effect is statistically different from zero at the 1 percent significance level. This effect is sizeable compared to the pre-period share of CalPERS patients being treated at ASCs of approximately, 74 percent. Additionally, the results indicate that physicians are, on average, more likely to treat both control and CalPERS patients at an ASC after the policy has been introduced. In fact, the share of patients treated at an ASC increases by approximately 7 percentage points, which is an increase of about 9.5 percent compared to the pre-period share of time spent at an ASC of 73 percent. This result could indicate that there are changes in the overall time-trend of where physicians treat patients. Alternatively, this result could suggest that there is a program spillover effect to the control group, which would bias the estimated treatment effects of the program, downward. This could occur if physicians spend a greater share of their work days at the ASC as a result of the policy and if control group patients have preferences for specific physicians.^20^

Also interesting, physicians are more likely to treat patients who are “sicker” as proxied by the patient Charlson Comorbidity score at a hospital. This result supports the idea that physicians treat the healthiest patients at ASCs, which may be justified for medical reasons.

### 6.3. Changing hours or changing schedules?

**Figure 2.**
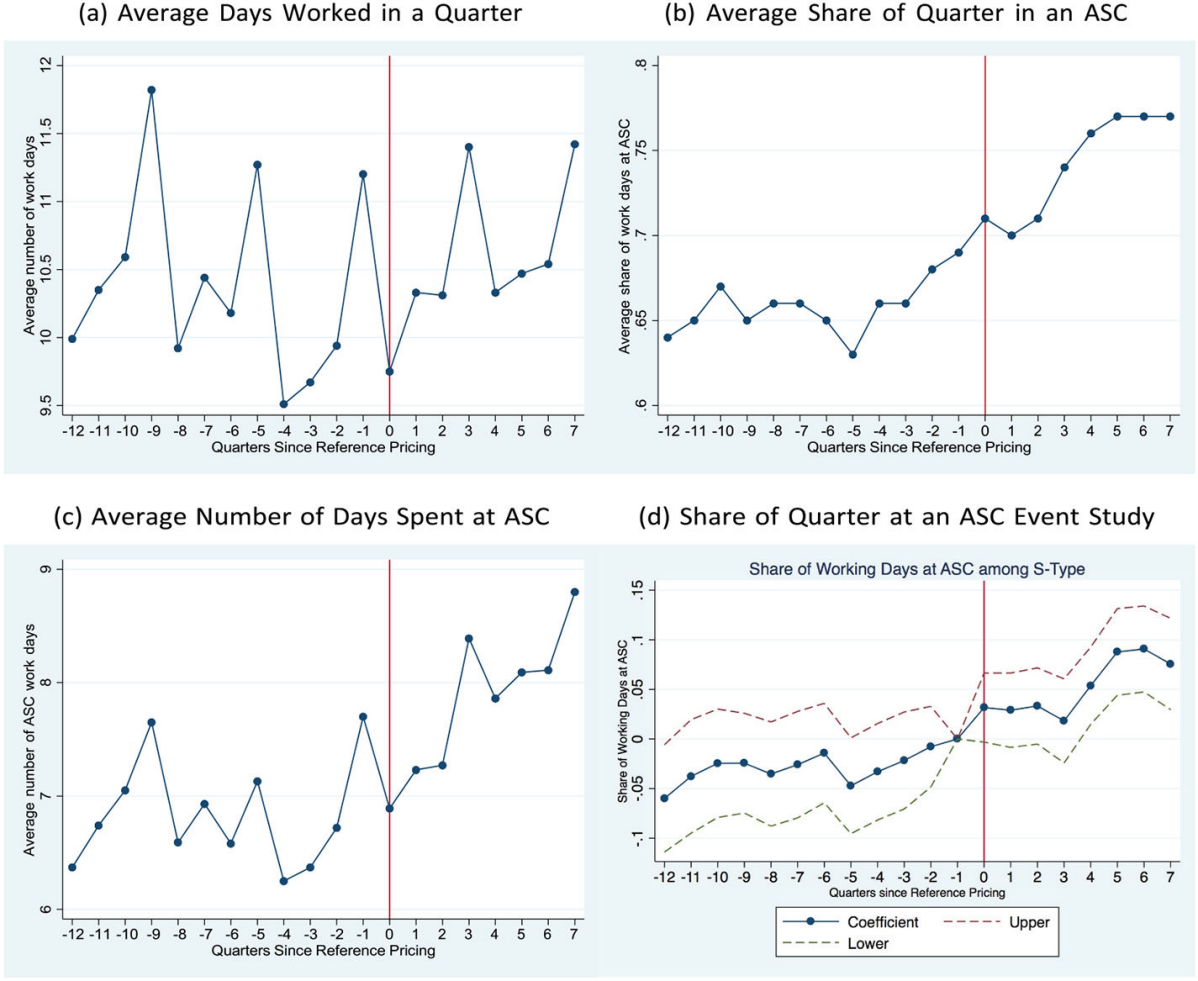
Work day variation among physicians. Figure 2a presents the average number of days worked in an ASC for physicians in the quarters since reference pricing. Figure 2b presents the average share of days worked at an ASC in a given quarter. Figure 2c presents the average number of days worked in an ASC in the quarters since reference pricing. Figure 2d presents estimates of the added share of working days spent at an ASC in a given quarter, in comparison to the quarter before the reform is introduced, which has been benchmarked to zero. Data is from colonoscopy patient who visits to Splitter physicians (i.e., physicians who work at both hospitals and Ambulatory Surgery Centers in 2011).

In order to better understand how physicians location changes are realized, I analyze whether physicians work more days in a given time period or if they work the same number of days, but just allocate their times differently across ASCS and hospitals. I am only able to observe the subset of days worked by a physician when she treats a CalPERS or control group patient. However, if CalPERS and control group patients represent a stable share of all patients seen by physicians and if they are also representative of the type of patients seen, inferring changes in physicians’ schedules from the data is possible. Figures 2(a-c) detail physicians’ work patterns over time. Figure 2(a) compares the average number of days worked within a quarter across the three physician types. There is cyclicity in the average number of days worked, with peaks tending to occur in the fourth quarter of any given year (i.e., quarters since reference pricing equal to −9,−5,−1,3,7) among physicians. This pattern appears to be consistent over time and does not seem to vary over the pre- and post-reference pricing period, which suggests that the number ofdays worked by physicians has not been impacted by the policy.

Figures 2(b,c) present the number of working days and share of working days spent at an ASC within a quarter for physicians. The figures both show that in the quarters leading up to the introduction of the policy, the number and share of work days spent at an ASC is relatively steady, although cyclical. However, after the policy is introduced, the number and share of work days spent at an ASC steadily increases over time, particularly in the fourth quarter after reference pricing is introduced. This is further supported by Figure 2(d) which presents estimates of the additional share of time physicians spend working at an ASC within a quarter, relative to the quarter prior to the introduction of the reform (i.e., Q4–2011).

Figures 2(b-d) indicate that physicians tend to spend a greater share of working days at ASCs after the policy is introduced. However, this response is not immediate and appears to take effect four quarters after the introduction of the policy. Specifically, approximately four quarters after the introduction of reference pricing, physicians spend between 5 and 10 percentage points more of their working days at an ASC. Assuming that physicians work an average of 40 hours per week, this implies an increase of 2 to 4 hours per week at the ASC. Relative to an initial share of days spent at ASCs of approximately 69 percent (the average observed in Q4–2011), this represents an increase of between 7 and 15 percent. Also, the initial lagged response observed prior to these quarters may be due to the time needed to re-establish work schedules since it may not be be possible for physicians to quickly change their schedules.

Physicians also tend to work, on average, at a similar number of ASCs before and after the policy (1.28 in the pre-period and 1.26 in the post-period). This implies that increased ASC affiliations are not driving the observed responses. Rather, the response appears to be driven by changes in physicians’ allocation of time at the ASCs at which they already work.

### 6.4. The importance of financial motives

I investigate if there is heterogeneity in physicians’ responses depending on their ASC ownership status to better understand the primitives underlying physicians’ movement to ASCs. In particular, I examine whether physicians who own an ASC are more responsive to the policy and more likely to treat CalPERS patients at ASCs than non-owners. This scenario is possible given that physicians who are owners of ASCs receive both a physician fee and part of the ASC facility’s profits when performing procedures at their own ASC.^21^ While this analysis is descriptive, it can provide suggestive evidence that there is a financial motive for location switching among physicians. This is consistent with the findings of Yee (2011) and Baker, Bundorf, and Kessler (2016), who show that pre-existing business relationships can influence physicians’ practice patterns.

To address this question, I determine physician ownership using the registry of accredited Outpatient Surgery Centers provided by the California Medical Board of California. Ownership status is also determined using documents provided by the State of California Business Search Results (California Business Search (2018)). Ownership is binary by definition (i.e., owner or non-owner) and is defined for each observed physician-ASC pairing since some physicians are observed working at multiple ASCs. I define a physician to be an owner of an ASC if during 2011, the physician is a part-owner, owner, or CEO of the ASC. If the physician is never observed working at an ASC for which they are an owner, they they are defined as an owner.^22^ Under this definition of ownership, approximately 69 percent of 2011 observations are from patient visits to owners and the remainder are from patient visits to non-owners.

I estimate Equation 1 separately for the owner and non-owner samples and also jointly, including both groups, using the following equation:

(3) $$1(ASC{)}_{{}_{ni}}=\delta_{{}_{0}}+\delta_{{}_{1}}\times Post_{{}_{i}}+\delta_{{}_{2}}\times Treat_{{}_{i}}\;\;+\delta_{{}_{4}}\times Post_{{}_{i}}\times Treat_{{}_{i}}+\delta_{{}_{5}}\times 1(Owner{)}_{{}_{i}}\times Treat_{{}_{i}}+\delta_{{}_{6}}\times 1(Owner{)}_{{}_{i}}\times Post_{{}_{i}}\;\;+\delta_{{}_{7}}\times 1(Owner{)}_{{}_{i}}\times Treat_{{}_{i}}\times Post_{{}_{i}}+X_{i}^{\prime }\delta +\alpha_{{}_{n}}+u_{{}_{ni}}$$

In Equation 3, 1(*Owner*)_*i*_ takes the value 1 if the *i*^*th*^ observation occurs with a physician who owns an ASC. 1(*Owner*)_*i*_ is zero if the *i*^*th*^ observation occurs with a physician who does not own an ASC. The other variables are as defined in Section 4. The main coefficient of interest is *δ*_7_. This coefficient indicates how much more likely a physician owner is to treat a CalPERS patient at an ASC than a non-owner, after reference pricing is introduced.

The results comparing owners to non-owners are presented in Table 4. The treatment effect estimate is similar to earlier estimates (6.6 percentage points), when owner and non-owner subsamples are separately analyzed in Columns 1 and 2. This indicates that both owners and owners are increasingly treating CalPERS patients at ASCs, relative to control group patients, after the introduction of the policy. Focusing on the results from the joint estimation, in Column 3, owners are much more likely to treat patients at an ASC in the post-period. In particular, owners are approximately 6.5 percentage points more likely to treat patients at an ASC in the post-period than in the pre-period. This represents an increase of approximately 9 percent compared to their share of time spent at an ASC in the pre-period. However, the coefficient estimate of *δ*_7_, is approximately zero and indicates that owners are not more responsive to the policy than non-owners in terms of their proclivity to treat CalPERS patients at ASCs after reference pricing.

This null result is likely driven by the fact that owners are increasingly treating *both* CalPERS and control group patients at the ASCs after the policy. This is in contrast to non-owners who only increase the treatment of CalPERS patients at ASCs. Reasons for the change in control-group trend among owners may be related to the reasons discussion in Results Section 6.1 (e.g., spillover effects, changes in control group demand for ASCs or physician time trends), but conclusively determining this is beyond the scope of this analysis.

### 6.5. Robustness checks

As a robustness check, the classification of “Splitter” physicians is alternatively defined. This is achieved by using the prior two years of a physician’s practice patterns, rather than one year. If there is inherent volatility in physicians’ work schedules, a two year look back period may provide a better understanding of their longer term practice trends. To do this, I re-estimate Equation 1 and limit the sample to physicians who are observed in at least all of 2010, 2011, 2012, and 2013. This is the case for approximately 72 percent of the sample. I then re-classify physicians as “Splitters” using the classification scheme discussed in Section 3, but now including observations from both 2010 and 2011 to do so. The results are presented in Table 5 and are qualitatively similar to the original results presented in Table 3. In particular, physicians are responsive to the policy as indicated by the estimated policy treatment effect of 5.8 percentage points versus an estimate of 6.4 obtained under the original physician classification scheme. Thus, the results are robust to the years that are used to classify “Splitter” physicians.

One could also worry that there is patient selection into solo-practicing physicians in a way that is related to the outcome. This is investigated by including observations belonging to physician groups as a robustness check. I then repeat the estimation of Equation 1. The results are presented in Table 6 of the Appendix. They largely mirror the main results presented in Table 3. For example, physicians’ treatment location changes in response to the policy. Specifically, the probability that a CalPERS patient is treated at an ASC when visiting a physician increases by 7.5 percentage points, relative to a control group patient. These results suggests that there is no loss of generality by focusing the analysis to solo-practicing physicians for whom type/practice patterns are more likely to be properly classified.

## 7. Conclusion

Given the growth in demand-side health insurance reforms, it is important to understand the responsiveness of physicians’ to such reforms. This is because of the important role physicians play in health care markets and the financial implications that their decisions have for patients. To understand this issue, this study analyzes whether physicians’ behavior changes after the introduction of a health insurance reform that only directly affects patients’ financial responsibility (i.e., cost-sharing). Specifically, I examine how physicians location decisions are affected by the introduction of reference pricing, which changes patients’ relative financial responsibility across medical locations (ASCs and hospitals).

Focusing on a panel of physicians observed before and after the policy and the location in which they provide medical treatment, this study finds that those physicians who are previously observed working across multiple locations (ASC and hospital) are responsive to the reform. After the policy has been introduced, the physicians are approximately 6.4 percentage points more likely to treat a CalPERS patient at the location that is now cheaper for them (ASC), relative to a control group patient. Additionally, introduction of the policy appears to be met with an increase in the number and share of days worked at an ASC among these physicians. However, there is no effect on the total number of days worked. Thus, physicians seem to reallocate their time toward ASCs.

To further understand the motives for physician movement across medical locations, I compare the response of physician owners and non-owners of ASCs. I do not find that owners are more likely to treat CalPERS patients at the ASCs compared to non-owners. However, this null result is largely driven by the increased share of control group patients who are treated at ASCs when visiting an owner physician; a pattern not found among non-owners.

Overall, the results imply that the behavior and actions of physicians are sensitive to less salient health insurance reforms that only affect a subset of patients. This is interesting because the availability of responsive and “amenable” physicians is necessary for the facilitation and implementation of demand-side health insurance reforms, such as this one. More specifically, the findings imply that changes to patient cost-sharing resulting from the introduction of a health insurance reform can affect physicians’ decisions, particularly where they deliver medical treatment. In a broader context, these results also imply that the response of physicians may be an important mechanism by which demand-side health insurance reforms successfully operate. Furthermore, the results are important for policymakers as they demonstrate that the ability for physicians to vary their treatment locations may be a source for reducing medical spending.

## Figures and Tables

**Table 1.** Data summary for physicians.

		Pre-Period (2009–2011)	Post-Period (2012–2013)
Pa:	Share Male	0.45	0.46
Pa:	Mean Age	52.77	52.97
Pa:	Share w/Charlson Index = 0	0.93	0.93
Ph:	Share Time at ASC	0.71	0.78
Ph:	Share Time at ASC (w/CalPERS patient)	0.72	0.85
Ph:	Share Time at ASC (w/control group patient)	0.71	0.77
Ph:	Share CalPERS patients	0.11	0.10
Ph:	Avg Physician fee ($)	517.23	511.17
Ph:	Avg Physician fee – ASC ($)	518.43	492.05
Ph:	Avg Physician fee – Hospital ($)	514.32	578.62
N		35,370	25,120

Note: The sample consists of colonoscopy patients who visit Splitter physicians (i.e., physicians who work at both hospitals and Ambulatory Surgery Centers in 2011). Data is limited to patient observations for those who visit physicians observed in all years of 2011, 2012 and 2013 and excludes physician groups. Data for 2009 and 2010 is included if it is available. Pa = Patient Characteristics; Ph = Physician Characteristics. “Avg Physician fee” refers to the average reimbursement that physicians receive per-procedure performed.

**Table 2.** Patient data summary statistics.

		Pre-Period (2009 – 2011)	Post-Period (2012 – 2013)
Pa:	Share Male	0.45	0.46
Pa:	Mean Age	52.77	52.97
Pa:	Share w/Charlson Index = 0	0.93	0.93
Ph:	Share Time at ASC	0.71	0.78
Ph:	Share Time at ASC (w/CalPERS patient)	0.72	0.85
Ph:	Share Time at ASC (w/control group patient)	0.71	0.77
Ph:	Share CalPERS patients	0.11	0.10
Ph:	Avg Physician fee ($)	517.23	511.17
Ph:	Avg Physician fee – ASC ($)	518.43	492.05
Ph:	Avg Physician fee – Hospital ($)	514.32	578.62
N		35,370	25,120

Note: The sample consists of colonoscopy patients who visit Splitter physicians (i.e., physicians who work at both hospitals and Ambulatory Surgery Centers in 2011). Data is limited to patient observations for those who visit physicians observed in all years of 2011, 2012 and 2013 and excludes physician groups.

**Table 3.** OLS difference-in-difference regression results where outcome is 1(*ASC*).

*Variable*	(1)
*Post*	0.071*** (0.014)
*Treat*	−0.011 (0.009)
*PostxTreat*	0.064*** (0.018)
1(Charlson = 1)	−0.054*** (0.008)
1(Charlson = 2	−0.058***(0.011)
1(Age 30–39 Yrs)	0.002 (0.015)
1(Age 40–49 Yrs)	0.010 (0.012)
1(50–59 Yrs)	0.010 (0.012)
1(60–64 Yrs)	0.001 (0.014)
1(Male)	0.007* (0.004)

Note: This table reports OLS coefficients from regressions where the outcome is 1(*ASC*). The sample consists of colonoscopy patients who visit Splitter physicians (i.e., physicians who work at both hospitals and Ambulatory Surgery Centers in 2011). Also note that: 1:

*** *p* ≤ 0.01

** *p* ≤ 0.05

* *p* < 0.10. 2: Standards errors are clustered at the physician-level-there are 213 clusters. Physician fixed effects are included.

**Table 4.** OLS regression results where outcome is 1(*ASC*).

	*Owners*	*Non-Owners*	*Owners & Non-Owners*
	(1)	(2)	(3)
*Post*	0.095*** (0.019)	0.030 (0.019)	0.030 (0.019)
*Treat*	−0.018* (0.010)	0.000 (0.013)	0.001 (0.013)
*Post* × *Treat*	0.066*** (0.022)	0.063** (0.029)	0.062** (0.029)
1(Owner) × *Post*			0.065** (0.027)
1(Owner) × *Treat*			−0.020 (0.016)
1(Owner) × *Post* × *Treat*			0.004 (0.037)
1(Charlson = 1)	−0.056*** (0.009)	−0.050*** (0.015)	−0.054*** (0.008)
1(Charlson = 2)	−0.069*** (0.014)	−0.038** (0.015)	−0.058*** (0.011)
1(Age 30–39 Yrs)	0.019 (0.019)	−0.026 (0.026)	0.002 (0.015)
1(Age 40–49 Yrs)	0.027 (0.018)	−0.016 (0.018)	0.011 (0.013)
1(Age 50–59 Yrs)	0.025 (0.017)	−0.014 (0.018)	0.011 (0.012)
1(Age 60–64 Yrs)	0.013 (0.019)	−0.018 (0.019)	0.002 (0.014)
1(Male)	0.008* (0.004)	0.006 (0.007)	0.007* (0.004)
N	37,023	23,467	60,490

Note: The sample consists of colonoscopy patients who visit Splitter physicians (i.e., physicians who work at both hospitals and Ambulatory Surgery Centers in 2011) that can be identified as owners or non-owners in the data. The table reports coefficient estimates where the outcome is 1(*ASC*). Also note that: 1:

*** *p* 0.01

** *p* 0.05

* *p* 0.10. 2: Standards errors are clustered at the physician-level; 120 clusters for owners; 93 clusters for non-owners; 213 clusters for owners and non-owners. 3: Physician fixed effects are included in all specifications.

## Footnotes

1 In contrast to earlier CalPERS reference pricing papers, Whaley and Brown (2018) study the pricing response of ASCs and hospitals to the reference pricing reform. They find modest price reductions among ASCs and some hospitals for facilities who are defined as having a “higher” exposure to CalPERS patients before reference pricing was implemented.

2 The share of CalPERS patients visiting a particular physician type is arguably even lower than that observed in the data. This is because the data analyzed includes all CalPERS patients exposed to reference pricing and non-CalPERS patients who have a particular PPO health plan; however, there are likely a number of other patients (i.e., those who do not have CalPERS or those do not have the particular PPO health plan) for a given physician.

3 Existing physicians refers to those physicians who are observed with patients before the health reform is introduced.

4 CalPERS is a public agency that manages the health and retirement benefits for over one million public employees as well as retired individuals. Approximately, 18 percent of active members who have health insurance through CalPERS are in a PPO plan (versus an HMO or Kaiser) (CalPERS (2017)).

5 Details of the policy are provided in the patient annual health benefits (Evidence of Coverage) book.

6 Reference pricing applied to facility fees, which generally cover the overhead cost of a facility. This is paid by the patient and the insurer. The other major billed fee is the physician’s fee which reimburses the physician and is also paid by both the patient and the insurer.

7 The analysis considers hospital outpatient departments but uses the term “hospitals.” Note: There can be cost-sharing limits for out-of-network ASCs.

8 Exemptions from the program are made for those CalPERS members who either present with a medical need to use a hospital or live more than 30 miles from an ASC.

9 However, features of the insurance plan, such as deductibles, may vary across patients.

10 This framework allows for the patient to initiate the discussion on where treatment should take place. This is particularly relevant in this specific medical context, colonoscopies – for diagnostic and screening purposes – which can be considered an elective medical procedure (CMS (2020)).

11 It is assumed that *E*[*ε^i^_ASC_*] = *E*[*ε^i^_Hospital_*] = 0

12 A physician group is identified as a physician entry that is listed with more than one physician name based on the 2011. I use the California Physician registry to search the name under the listed physician to ensure that it is associated with a single physician.

13 If labor contracts are negotiated annually, it is ideal to capture the most recent labor circumstance as reflected by the year closest to reference price implementation.

14 The robustness of this specification is further checked in Section 6.5.

15 For example, a Charlson Comorbidity score of zero means that the patient has no other comorbid conditions/diseases.

16 A physician, here, is defined as either a physician group or a solo-practicing physician.

17 Information was provided during the open enrollment period.

18 *X* is a vector of patient covariates that includes a patient’s age in 10 year bins (except 18–29 and 60–64 years), a binary indicator for whether they are male, the Charlson Comorbidity Index (Charlson et al. (1987)) of the patient based on pre-period Current Procedural Terminology (CPT) medical codes, and indicators for the health care referral region of the patient.

19 Approximately 85 percent of all patients visit a physician (group practice or solo-practice) who was observed in all years between 2011–2013.

20 In principal, control group patients may be amenable to using an ASC because ASCs are generally less expensive (Munnich and Parente (2014)).

21 The analysis considered assumes that there are no binding capacity constraints on the use of ASCs by either owners or non-owners.

22 For patients who are treated by physicians at ASCs in 2011, I am able to identify whether the physician owns the ASC for approximately 82 percent of observations.

## Appendix

**Table A1.** Robustness check – OLS difference-in-difference regression results where outcome is 1(*ASC*) and physician type is defined using two years of data.

*Variable*	(1)
*Post*	0.061*** (0.015)
*Treat*	−0.010 (0.009)
*PostXTreat*	0.058*** (0.018)
1(Charlso*n*=1)	−0.049*** (0.009)
1(Charlso*n*=2)	−0.055*** (0.011)
1(Age 30–39Yrs)	−0.002 (0.014)
1(Age 40–49Yrs)	0.006 (0.012)
1(50–59Yrs)	0.008 (0.012)
1(60–64Yrs)	−0.002 (0.013)
1(Male)	0.007* (0.004)
N	61,388

Note: This table reports coefficient estimates where the outcome is 1(*ASC*). The sample consists of colonoscopy patients who visit Splitter physicians (i.e., physicians who work at both hospitals and Ambulatory Surgery Centers in 2010 and 2011). Also note that: 1:

*** *p*0.01

** *p*0.05

* *p*0.10.

: Standards errors are clustered at the physician-level – there are 215 clusters.3: Physician fixed effects are also included.

**Table A2.** Robustness check – OLS Difference-in-difference regression results where outcome is 1(*ASC*) and physician groups are included.

*Variable*	(1)
*Post*	0.067*** (0.014)
*Treat*	0.007 (0.009)
*PostxTreat*	0.075*** (0.018)
1(Charlso*n*=1)	−0.062*** (0.007)
1(Charlso*n*=2)	−0.063*** (0.008)
1(Age 30–39Yrs)	0.019 (0.015)
1(Age 40–49Yrs)	0.026* (0.015)
1(50–59Yrs)	0.025* (0.015)
1(60–64Yrs)	0.018 (0.014)
1(Male)	0.006* (0.003)
N	113,106
N	60,490

Note: This table reports OLS coefficients from regressions where the outcome is 1(*ASC*). The sample consists of colonoscopy patients who visit Splitter physicians (i.e., physicians who work at both hospitals and Ambulatory Surgery Centers in 2011). Also note that: 1:

*** *p*≤0.01

** *p*≤0.05

* *p*≤0.10. 2: Standards errors are clustered at the physician-level – there are 283 clusters. 3: Physician fixed effects are also included.
